# Preclinical characterization of the Omicron XBB.1.5-adapted BNT162b2 COVID-19 vaccine

**DOI:** 10.1038/s41541-024-01013-9

**Published:** 2024-11-20

**Authors:** Kayvon Modjarrad, Ye Che, Wei Chen, Huixian Wu, Carla I. Cadima, Alexander Muik, Mohan S. Maddur, Kristin R. Tompkins, Lyndsey T. Martinez, Hui Cai, Minah Ramos, Sonia Mensah, Brittney Cumbia, Larissa Falcao, Andrew P. McKeen, Jeanne S. Chang, Kimberly F. Fennell, Kevin W. Huynh, Thomas J. McLellan, Parag V. Sahasrabudhe, Wei Chen, Michael Cerswell, Miguel A. Garcia, Shilong Li, Rahul Sharma, Weiqiang Li, Kristianne P. Dizon, Stacy Duarte, Frank Gillett, Rachel Smith, Deanne M. Illenberger, Kari Sweeney Efferen, Annette B. Vogel, Annaliesa S. Anderson, Uğur Şahin, Kena A. Swanson

**Affiliations:** 1 Vaccine Research and Development, Pfizer Inc., Pearl River, NY USA; 2 Viral Vaccines, Vaccine Research and Development, Pfizer Inc., Pearl River, NY USA; 3 Discovery Sciences, Pfizer Inc., Groton, CT USA; 4 BioNTech SE, Mainz, Germany; 5 Global Biometrics and Data Management, Pfizer Inc., Pearl River, NY USA; 6 Early Bioprocess Development, Vaccine Research and Development, Pfizer Inc., Pearl River, NY USA

**Keywords:** RNA vaccines, SARS-CoV-2

## Abstract

As SARS-CoV-2 evolves, increasing in potential for greater transmissibility and immune escape, updated vaccines are needed to boost adaptive immunity to protect against COVID-19 caused by circulating strains. Here, we report features of the monovalent Omicron XBB.1.5-adapted BNT162b2 vaccine, which contains XBB.1.5-specific sequence changes, relative to the original BNT162b2 backbone, in the encoded prefusion-stabilized SARS-CoV-2 spike protein (S(P2)). Biophysical characterization of Omicron XBB.1.5 S(P2) demonstrated that it maintains a prefusion conformation and adopts a flexible, predominantly open, state, with high affinity for the human ACE-2 receptor. When administered as a 4th dose in BNT162b2-experienced mice, the monovalent Omicron XBB.1.5 vaccine elicited substantially higher serum neutralizing titers against pseudotyped viruses of Omicron XBB.1.5, XBB.1.16, XBB.1.16.1, XBB.2.3, EG.5.1 and HV.1 sublineages and phylogenetically distant BA.2.86 lineage than the bivalent Wild Type + Omicron BA.4/5 vaccine. Similar trends were observed against Omicron XBB sublineage pseudoviruses when the vaccine was administered as a 2-dose series in naive mice. Strong S-specific Th1 CD4^+^ and IFNγ^+^ CD8^+^ T cell responses were also observed. These findings, together with real world performance of the XBB.1.5-adapted vaccine, suggest that preclinical data for the monovalent Omicron XBB.1.5-adapted BNT162b2 was predictive of protective immunity against dominant SARS-CoV-2 strains.

## Introduction

The evolution of Severe Acute Respiratory Syndrome Coronavirus-2 (SARS-CoV-2), the cause of coronavirus disease 2019 (COVID-19), has been marked by sustained periods of genetic and antigenic drift, best exemplified by the continual emergence of new variants since the appearance of the Omicron variant of concern (VOC) in November 2021^1^. The initial antigenic shift to Omicron BA.1, followed by the dominance of Omicron BA.4/5, prompted updates to COVID-19 vaccines to better match prevalent circulating virus strains. Bivalent formulations of the BNT162b2 vaccine, encoding the spike (S) protein of the Wuhan-Hu-1 wild type (WT) strain (GenBank MN908947.3) and either Omicron BA.1 (GISAID EPI_ISL_8880082) or BA.4/5 (GISAID EPI_ISL_15030644) sublineages, subsequently demonstrated effectiveness against COVID-19 in the season after their introduction^2–5^. The later emergence of recombinant Omicron XBB sublineages, which dominated the epidemiologic landscape throughout 2023, showed that SARS-CoV-2 is able to evolve toward greater transmissibility and to occupy pockets of antigenic space that evade previously established host immunity^6^. The Omicron XBB.1.5 sublineage exhibits greater antigenic distance from Omicron BA.1 than the latter does from the WT strain^3,7^. Waning immunity conferred by prior vaccination or infection with XBB sublineages and the ineffectiveness of nearly all licensed monoclonal antibody therapies against XBB.1.5^8^ reflect this immunologic trend^9,10^. As such, updating COVID-19 vaccines to more closely matched circulating strains is essential to boosting relevant immunity and maintaining effectiveness against a range of clinical outcomes. Accumulating evidence shows that this principle, well-established for vaccines against influenza and other pathogens, also applies to COVID-19 vaccines^9,10^.

BNT162b2 RNA encodes the full-length (FL) S protein stabilized in the prefusion conformation through the substitution of amino acid (aa) positions 986 and 987 to proline residues (S(P2))^11–14^, a modification that has increased the antigen’s immunogenicity and expression, as compared to the postfusion state^15^. To address the increasing dominance of the antigenically distant Omicron XBB sublineages, we modified the original COMIRNATY^®^ vaccine—using the same mRNA backbone as the BNT162b2 that encoded WT S(P2)—to encode an Omicron XBB.1.5 FL S(P2). As the structure of the Omicron XBB.1.5 S(P2) has not been resolved, we sought to characterize the structural and biophysical properties of the mRNA encoded prefusion-stabilized S on this strain-adapted background; including its thermostability profile, human angiotensin converting enzyme-2 (ACE-2) receptor affinity, glycosylation pattern and overall structure and receptor binding domain (RBD) conformational dynamics.

Omicron XBB.1.5-adapted BNT162b2 vaccine formulations were also evaluated in preclinical immunogenicity studies in vaccine-experienced and naïve mice and included the assessment of neutralizing antibody responses against a panel of pseudoviruses of varying phylogenetic proximity and measurement of antigen-specific CD4^+^ and CD8^+^ T cell responses. These studies sought to inform the optimal vaccine valency and composition for eliciting protective immunity. The data presented here provide a basis for understanding the key biophysical and immunologic features of an Omicron XBB.1.5-adapted vaccine in the dynamic landscape of SARS-CoV-2.

## Results

### Omicron XBB.1.5 S(P2) biophysical and structural characterization

**Fig. 1:**
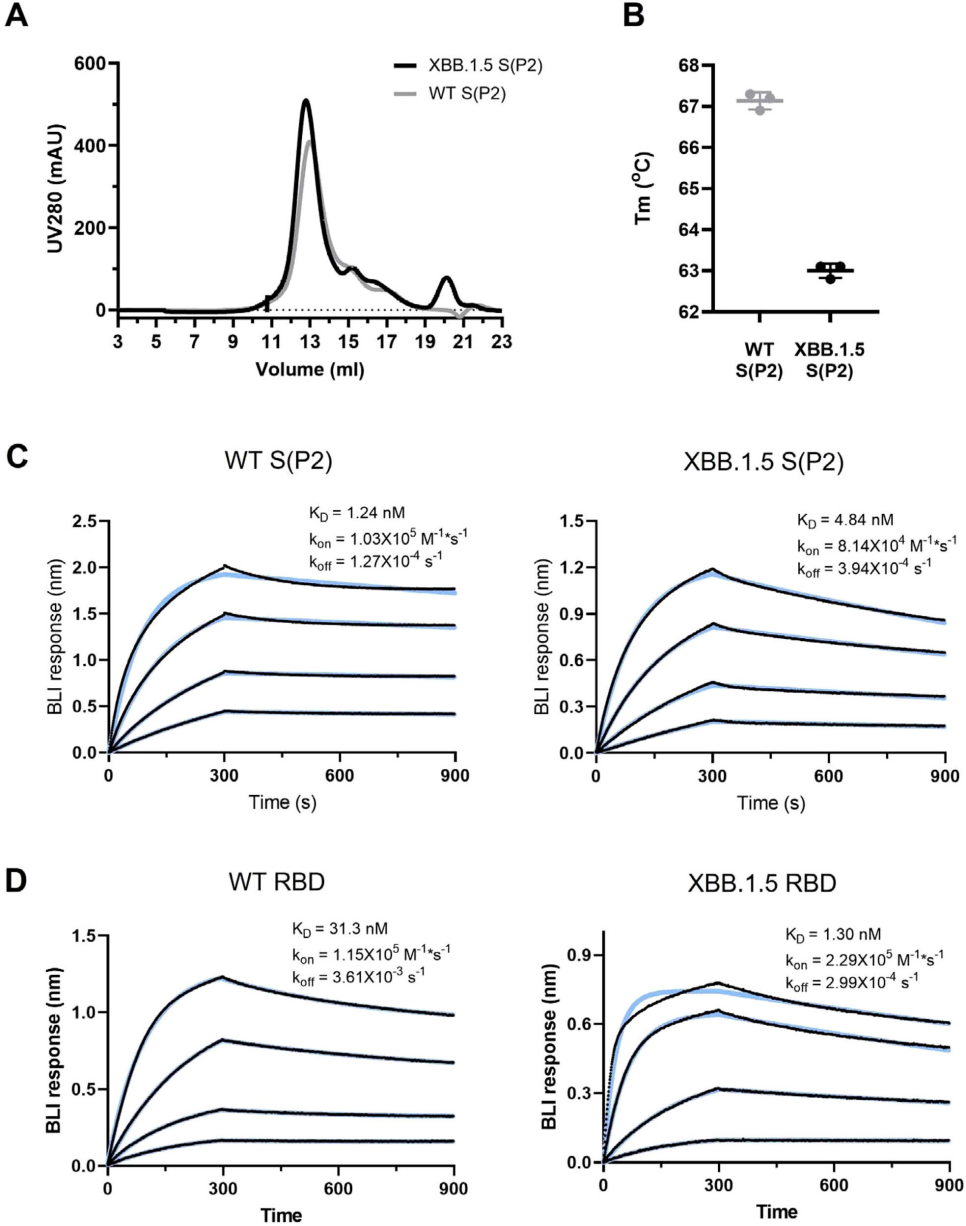
Biophysical properties and ACE-2 receptor binding affinities SARS-CoV-2 WT and Omicron XBB.1.5 FL S(P2) and RBD. **A** Size exclusion chromatography (SEC) profile of the purified WT and Omicron XBB.1.5 FL S(P2) proteins equivalent to the protein antigen encoded by the BNT162b2 vaccines. **B** Melting temperature (Tm) of DDM-purified S(P2) proteins at 0.35 mg/mL concentration. Assay was run in triplicate. Biolayer interferometry (BLI) sensorgram showing binding of (**C**) purified S(P2) proteins and (**D**) RBD to immobilized human angiotensin converting enzyme-2 peptidase domain (ACE-2-PD). Binding data are in black; 1:1 binding model fit to the data is in color. Apparent kinetic parameters are provided in the graph. K_D_ = equilibrium dissociation constant; k_on_ = binding rate constant; k_off_ = dissociation rate constant.

The S(P2) antigen of Omicron XBB.1.5 was expressed from DNA corresponding to the XBB.1.5-adapted BNT162b2 RNA coding sequence using similar methods, as previously reported^13^. The sequence of the DNA expressed S(P2) was the same as that encoded in the BNT162b2 mRNA. After affinity purification, XBB.1.5 S(P2) eluted as a single peak by size exclusion chromatography (SEC), similar to the WT S(P2) (Fig. 1A). Peak fractions of XBB.1.5 S(P2) mostly contained cleaved S1 and S2 subunits, as was also observed for WT S(P2) (Supplementary Fig. 1)^13^. The SEC peak fraction was then assayed by thermal shift assay (TSA) and biolayer interferometry (BLI). The XBB.1.5 S(P2) had a melting temperature (T_m_) of 63.0 ± 0.2 °C, approximately 4 °C lower than the T_m_ of the WT S(P2) (*n* = 3) (Fig. 1B). XBB.1.5 S(P2) bound to the human ACE-2 peptidase domain (ACE-2-PD) with an affinity (K_D_ 4.84 nM) that was slightly less potent than that observed for WT S(P2) (K_D_ 1.24 nM), primarily due to the faster binding off rate of XBB.1.5 S(P2) (Fig. 1C). The binding affinity of purified monomeric RBD to ACE-2-PD binding affinity was also measured. In this case, XBB.1.5 RBD exhibited an affinity (K_D_ 1.30 nM) that was 24-fold more potent than that observed for WT RBD (K_D_ 31.3 nM).

Purified XBB.1.5 S(P2) was analyzed by liquid chromatography mass spectrometry (LCMS) to identify N-linked glycosylation sites. Twenty-seven N-linked glycosylation sites were detected in S(P2) over a total protein sequence coverage of 92%. The glycosylation pattern was generally similar to that observed for WT S(P2)^16^ (Supplementary Fig. 2). However, several new glycosylation sites were also identified in XBB.1.5 S(P2), including N164, N536, N824, N856, N907 and N1119. Two glycosylation sites, N17 and N282, that were reported in WT S(P2)^16^, were not detected in XBB1.5 S(P2).

**Fig. 2:**
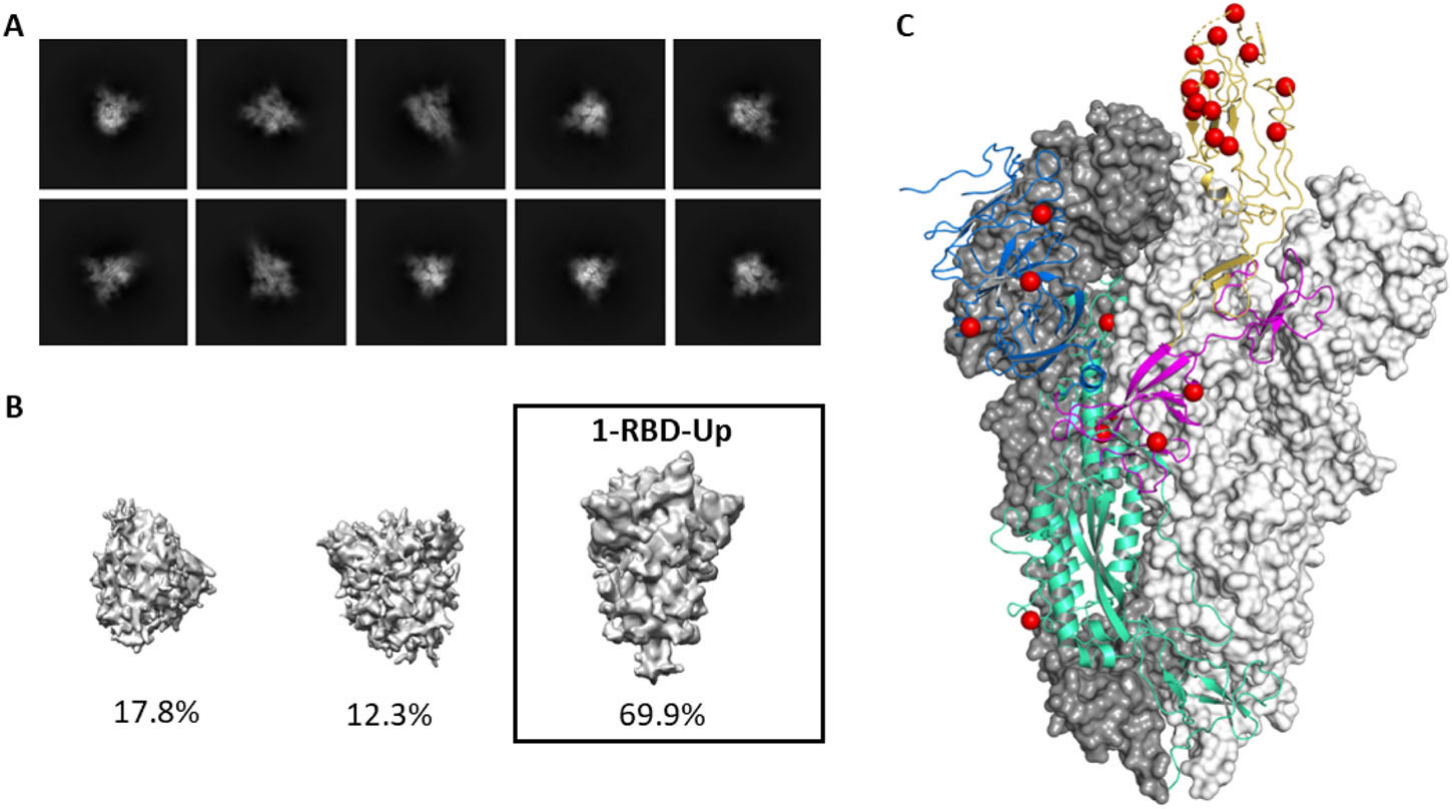
Cryo-EM structure of SARS-CoV-2 Omicron XBB.1.5 spike protein. **A** Representative 2D class averages of full-length prefusion stabilized Omicron XBB.1.5 S(P2). Box size is 40.5 nm in each dimension. **B** Maps from ab initio reconstruction reveal only one class resembling the S(P2) protein particles with 1-RBD in the ‘up’ position. These particles were used for the final reconstruction. Percentages of the particle population represented in each class are indicated below the models. **C** The overall structure of Omicron XBB.1.5 S(P2) trimer modeled based on the 2.98 Å density map. Two of the three protomers with RBD in a ‘down’ conformation are represented by a molecular surface colored in white and grey. The remaining protomer with RBD in an ‘up’ conformation is represented by a ribbon diagram; The NTD is colored blue; the RBD is colored yellow; the remaining S1 subunit is colored purple; and the S2 subunit is colored green. Amino acid residues that differ between Omicron XBB.1.5 and the ancestral strain are represented by red spheres.

The structure of purified XBB.1.5 S(P2) was resolved by cryogenic electron microscopy (cryo-EM). Two-dimensional (2D) classification of particles from cryo-EM data revealed a particle population that closely resembled the prefusion conformation of WT S (Fig. 2). Processing and refinement of the dataset (Supplementary Fig. 3) yielded a high-quality three-dimensional (3D) map with a nominal resolution of 2.98 Å (Fig. 2, Supplementary Table 1), into which a previously published atomic model (PDB ID: 7TGW) was fitted and rebuilt. The structure revealed that prefusion S(P2) in a 1-RBD-up conformation accounted for the majority (70%) of the high-resolution particles, contrasting with the WT S(P2) all-RBD-down (79.6%) conformation^13^. The diminished resolution of the RBD-up conformation, as compared to the other parts of the S structure, suggests a conformational flexibility and dynamic equilibrium between RBD ‘up’ and RBD ‘down’ states that is consistent with other reports of SARS-CoV-2 Omicron S structures^17,18^. This resolved structure of XBB.1.5 S(P2), therefore, closely resembles the more open form and flexibility of the S protein of earlier Omicron sublineages^19^.

### BNT162b2 Omicron XBB.1.5 immunogenicity

#### Humoral immune response—booster vaccination

Omicron-adapted BNT162b2 formulations were evaluated in two murine studies that varied by prior immune exposure (Supplementary Fig. 4). In a booster study, female BALB/c mice were experienced with two doses of the monovalent WT BNT162b2 vaccine on Day 0 and Day 21, followed by a single dose of the BNT162b2 bivalent WT + Omicron BA.4/5 vaccine three months later (Supplementary Fig. 4A). This regimen approximates the relevant immune background of the vaccinated human population that was exposed to S of the ancestral strain and Omicron lineage through vaccination. One month later, animals received one of four BNT162b2 vaccine formulations: monovalent BA.4/5, bivalent WT + BA.4/5, monovalent XBB.1.5 or bivalent XBB.1.5 + BA.4/5. Sera were collected prior to and one month after the administration of the last dose for assessment of pseudovirus neutralization; splenocytes were collected one month after the last dose to assess T cell responses.

**Fig. 3:**
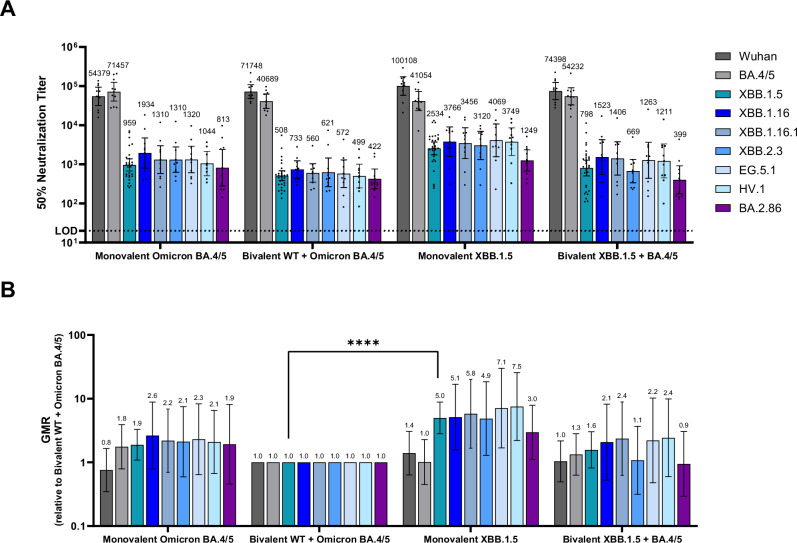
Pseudovirus neutralization titers (NT_50_) elicited by BNT162b2 variant-adapted vaccines administered as a 4th dose in immune-experienced mice. Female BALB/c mice (10/group) that were previously vaccinated (per schedule described in Supplementary Fig. 4A) with two-doses of monovalent original WT BNT162b2, and one subsequent dose of bivalent WT + Omicron BA.4/5 received a single intramuscular dose of one of these vaccine regimens: monovalent Omicron BA.4/5, bivalent WT+ Omicron BA.4/5, monovalent Omicron XBB.1.5 or bivalent Omicron BA.4/5 + Omicron XBB.1.5. All vaccine formulations contained a total dose of 0.5 µg. Fifty-percent geometric mean serum neutralizing titers were characterized in a pseudovirus neutralization assay at one-month post-4th dose against the WT reference strain, the Omicron sublineages BA.4/5, XBB.1.5, XBB.1.16, XBB.1.16.1, XBB.2.3, EG.5.1, HV.1 and the lineage BA.2.86. **A** 50% pseudovirus neutralization titers are shown as geometric mean titers (GMT) ± 95% CI of 10 mice per vaccine group. Each point represents an individual animal. **B** The geometric mean ratio (GMR) is the GMT of individual pseudovirus responses of each vaccine group (monovalent Omicron BA.4/5, monovalent Omicron XBB.1.5 or bivalent Omicron BA.4/5 + Omicron XBB.1.5) divided by the GMT of analogous pseudovirus responses of the bivalent WT + Omicron BA.4/5 group. Statistical differences were analysed by ANOVA with a Dunnett’s multiple comparison test (****p < 0.0001). The limit of detection (LOD) is the lowest serum dilution, 1:20.

The fifty percent neutralization geometric mean titers (GMTs) at one-month post-4th dose were substantially different across the vaccine groups (Fig. 3A). Neutralizing activity against XBB.1.5 and other circulating XBB sublineages (XBB.1.16, XBB.1.16.1, XBB.2.3, EG.5.1 and HV.1) was highest among animals that received the monovalent XBB.1.5 booster, particularly compared to the bivalent WT + BA.4/5 group. GMT values were similar across the XBB sublineages tested in the group that received the monovalent XBB.1.5 vaccine. Overall, the post-boost GMTs elicited by the monovalent XBB.1.5 vaccine against XBB sublineages were five-to-eight-fold higher than those elicited by the bivalent WT + BA.4/5 vaccine (*p* < 0.05), while the response against the BA.2.86 lineage was three-fold higher in the XBB.1.5 vaccine group (Fig. 3A, B), though not statistically significant (*p* = 0.05). Two versions of BA.2.86 pseudoviruses were generated and tested because of the variability in spike sequences from early isolates^20^. These two pseudoviruses, which differed by one amino acid substitution in the subdomain of the S1 subunit (I670V), were equally sensitive to neutralization. As such, data shown for BA.2.86 in Fig. 3 represent the current consensus sequence that does not contain the I670V mutation. Overall, neutralizing antibody responses were highest against WT and BA.4/5 irrespective of the vaccine group, reflective of prior exposures to WT and BA.4/5 S.

Pre-boost, baseline GMTs (pre-4th vaccine dose) varied some across groups (Supplementary Fig. 5); however, the trends across different pseudovirus lineages between groups were similar. The fold rise in geometric mean neutralizing titers (GMFR) from pre- to post-4th dose were calculated for each of the three lineages tested. The GMFRs in neutralizing activity against XBB.1.5 pseudovirus from the pre- to post-boost time points were highest in the monovalent XBB.1.5 and bivalent XBB.1.5 + BA.4/5 vaccine groups (GMFR 17.5 and 12.5, respectively) followed by the monovalent BA.4/5 vaccine group (GMFR 10.1) (Supplementary Fig. 6).

#### Humoral immune response—primary series vaccination

**Fig. 4:**
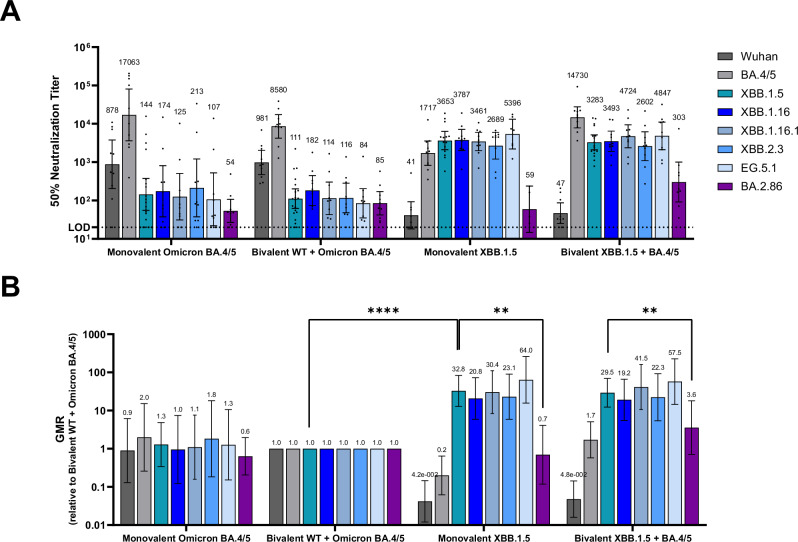
Pseudovirus neutralization titers (NT_50_) elicited by BNT162b2 variant-adapted vaccines administered as a primary series in naive mice. Female BALB/c mice (10/group) vaccinated with two-doses of one of the following vaccine regimens at a twenty-one-day interval: monovalent Omicron, BA.4/5, bivalent WT + Omicron BA.4/5, monovalent Omicron XBB.1.5 or bivalent Omicron BA.4/5 + Omicron XBB.1.5. All vaccine formulations contained a total dose of 0.5 µg. Serum neutralizing antibody responses were measured by a pseudovirus neutralization assay at one-month post-second dose against the WT reference strain, the Omicron sublineages BA.4/5, XBB.1.5, XBB.1.16, XBB.1.16.1, XBB.2.3, EG.5.1 and the lineage BA.2.86. **A** 50% pseudovirus neutralization titers are shown as geometric mean titers (GMT) ± 95% CI of 10 mice per vaccine group. Each point represents an individual animal. **B** The geometric mean ratio (GMR) is the GMT of individual pseudovirus responses of each vaccine group (monovalent Omicron BA.4/5, monovalent Omicron XBB.1.5 or bivalent Omicron BA.4/5 + Omicron XBB.1.5) divided by GMTs of analogous pseudovirus responses of the bivalent WT + Omicron BA.4/5 group. Statistical differences were analysed by ANOVA with a Dunnett’s multiple comparison test (**p < 0.01; ****p < 0.0001). The limit of detection (LOD) is the lowest serum dilution, 1:20.

In a primary series study the same vaccine formulations used in the booster study were administered on Days 0 and 21 in naive female BALB/c mice (Supplementary Fig. 4B). Sera collected one month after the second dose were tested against the same pseudovirus panel used in the booster study. Similar to the findings from the booster study, the monovalent XBB.1.5 vaccine elicited substantially higher neutralizing titers against all tested XBB sublineages (XBB.1.5, XBB.1.16, XBB.1.16.1, XBB.2.3 and EG.5.1) (Fig. 4A, B) compared to the bivalent WT + BA.4/5 vaccine, though at a much higher magnitude of difference than observed in the booster study (*p* < 0.01). Responses were similar across the XBB pseudoviruses in the monovalent XBB.1.5 vaccine group, with the exception of BA.2.86 which had a significantly lower GMR compared to XBB.1.5 (*p* < 0.05). The BA.2.86 pseudovirus also escaped neutralization elicited by the bivalent XBB.1.5 + BA.4/5 vaccine group (*p* < 0.05), compared to XBB.1.5 GMR (Fig. 4B), despite BA.2.86 neutralizing responses being an order of magnitude higher than in the other groups (Fig. 4A).

#### Cellular immune response

**Fig. 5:**
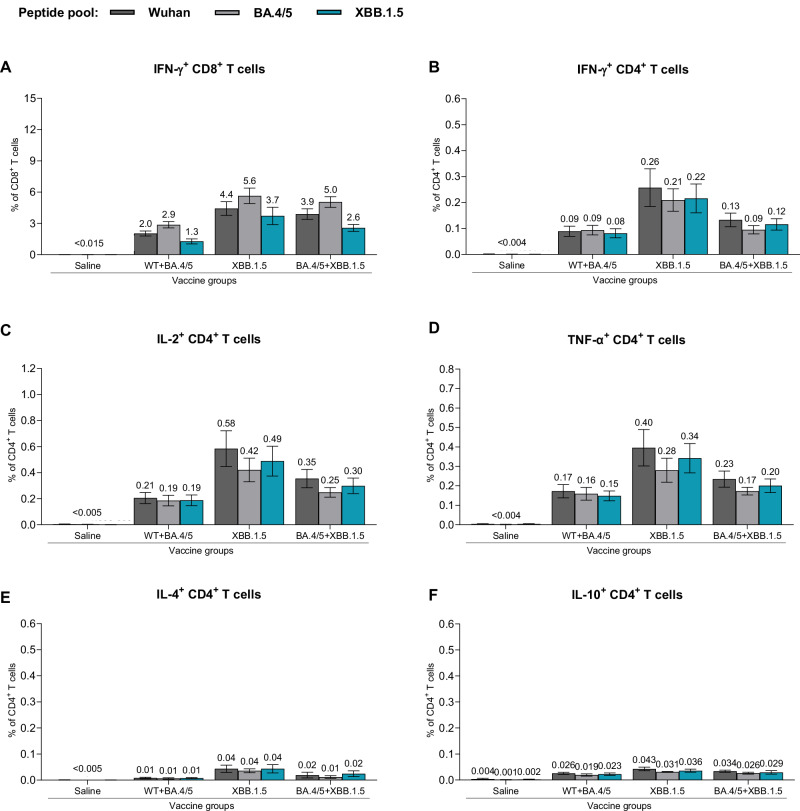
T cell responses elicited by BNT162b2 variant-adapted vaccines administered as a 4th dose in BNT162b2-experienced mice. One-month after the 4th dose, S-specific CD4^+^ and CD8^+^ splenocytes (*n* = 5/group) were characterized by a flow cytometry-based intracellular cytokine staining assay. All samples were stimulated separately with S peptide pools from the WT reference strain, Omicron BA.4/5, or XBB.1.5 sublineages. Graphs show the frequency of CD8^+^ T cells expressing IFN-γ (**A**) and the frequency of CD4^+^ T cells expressing IFN-γ (**B**), IL-2 (**C**), TNF-α (**D**), IL-4 (**E**) and IL-10 (**F**) in response to stimulation with each peptide pool across vaccine groups. Bars depict mean frequency ± SEM.

**Fig. 6:**
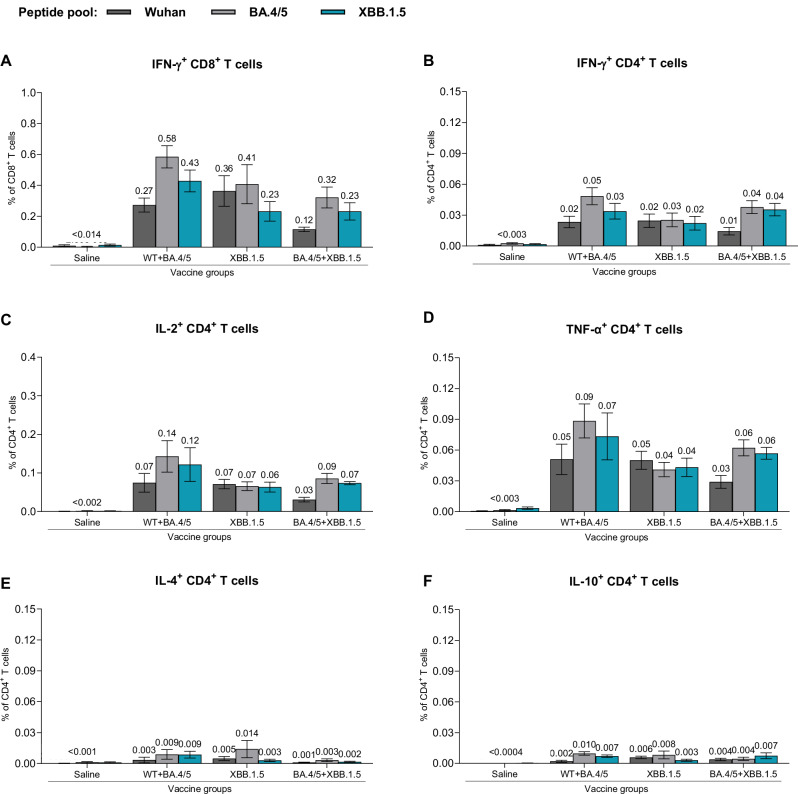
T cell immune responses elicited by BNT162b2 variant-adapted vaccines administered as a primary series in naive mice. At one-month post-second dose (completion of primary series), S-specific T cells from fresh spleens (*n* = 5) were measured by intracellular cytokine staining assay. All samples were stimulated separately with S peptide pools from the WT reference strain and the Omicron BA.4/5 and XBB.1.5 sublineages. Graphs show the frequency of CD8^+^ T cells expressing IFN-γ (**A**) and the frequency of CD4^+^ T cells expressing IFN-γ (**B**), IL-2 (**C**), IL-4 (**D**), TNF-α (**E**) and IL-10 (**F**) in response to stimulation with each peptide pool across vaccine groups. Bars depict mean frequency ± SEM.

T cell responses were measured following XBB.1.5-adapted vaccine administration in both the booster and primary series dosing regimens. Spleens collected one month following the last vaccine dose were analyzed for frequencies of S-specific T cells, using a flow cytometry-based intracellular cytokine staining (ICS) assay (Supplementary Fig. 7). Splenocytes were stimulated with S peptide pools representing the amino acid sequence of the WT strain or the Omicron BA.4/5 and XBB.1.5 sublineages. In the booster study, all vaccine formulations induced high frequencies of S-specific CD4^+^ and CD8^+^ T cells, with a trend toward slightly higher responses in the monovalent XBB.1.5 vaccine group (Fig. 5). The magnitude of IFN-γ-producing T cell responses was higher for CD8^+^ T cells than for CD4^+^ T cells (Fig. 5A, B). The frequency of IL-2- and TNF-α-expressing CD4^+^ T cells trended slightly higher than the frequency of CD4^+^ T cells producing IFN-γ (Fig. 5B–D). Very low levels of IL-4- and IL-10-expressing CD4^+^ T cells were observed (Fig. 5E, F), thus supporting a Th1-biased response profile that was consistent with prior preclinical and clinical data for BNT162b2^13,21^. T cell responses in the primary series study were similar to those in the booster study, despite the overall magnitude of responses being lower (Fig. 6A–E). Notably, the magnitude of T cell response to each of the strains (WT, Omicron BA.4/5 and Omicron XBB.1.5) was similar within each vaccine group in both booster and primary series studies. These results suggest that polyclonal T cell responses are maintained in mice after primary or booster vaccination and are not significantly impacted by the mutational differences of the XBB.1.5 sublineage as compared to earlier strains.

## Discussion

The evolution of SARS-CoV-2 has prompted an adaptive approach to the continued development of updated vaccines to maintain optimal protection against COVID-19. Since early 2020, when public health crises were declared by multiple national agencies and international normative authorities^22^, more than 4200 SARS-CoV-2 unique lineages and sublineages have been identified^23^. Despite this large genetic diversity, few strains have gained significant advantage to successfully dominate the epidemiologic landscape for extended periods. The most recent strains to exceed a global proportion of 50% are the recombinant Omicron XBB.1.5 sublineage and the Omicron BA.2.86 derivative JN.1 lineages, to include those that have acquired amino acid substitutions in the S protein, such as S31del, R346T, F456L, Q493E, that have conferred improved viral fitness and consequent growth advantage (i.e., KP.2, LB.1, KP.3, KP.3.1.1).

For nearly a year, Omicron XBB sublineages and their derivatives accounted for the overwhelming majority of new infections globally^24^. The descendants of this recombinant lineage cluster consistently exhibited significant immune escape from approved monoclonal antibodies^8,25,26^ The large antigenic distance of these sublineages from earlier SARS-CoV-2 strains, together with waning effectiveness of earlier vaccine iterations based on strains that are no longer circulating and the induction of a more broadly relevant polyclonal antibody response, necessitated updates to the COVID-19 vaccine.

In the current report, the preclinical data demonstrate an immune response profile that supported the Omicron XBB.1.5 vaccine update. Additionally, for the first time, the trimeric prefusion stabilized structure of Omicron XBB.1.5 S has been resolved. Biophysical characterization studies demonstrate that the Omicron XBB.1.5-adapted BNT162b2 vaccine encodes an S(P2) that authentically presents an antigenically favorable prefusion conformation and ACE-2 binding site. XBB.1.5 S(P2), despite having many mutations, contains biophysical features that are remarkably similar to the S protein of the ancestral WT strain. However, the RBD in Omicron XBB.1.5 S(P2) conforms to a more open and flexible state that contrasts with the closed state of the ancestral strain and early Omicron lineages^27^. A cryo-EM structure of the BA.2.86 spike was recently reported and showed it adopted a more closed, all-RBD-down conformation that reverts more toward the WT S^28^. Prior reports have demonstrated that the S of the Omicron BA.2 sublineage, of which XBB.1.5 is a recombinant, may be more compact and thermostable than other variants^29^. The Omicron XBB.1.5 RBD has an ACE2 binding affinity that is substantially more potent than its ancestral counterpart (Fig. 1D). The increased ACE2 potency likely results from mutations G446S (introduce H-bond with Q42^ACE2^), Q498R (introduce salt bridge with D38^ACE2^), N501Y (introduce π-stacking with Y41^ACE2^), and Y505H (introduce salt bridge with E37^ACE2^) acquired by the XBB.1.5 RBD in the receptor binding motif (RBM). However, when ACE2 binding was assessed in the context of the full-length trimeric S(P2), the Omicron XBB.1.5 S(P2) exhibited a lower binding affinity than the WT S(P2), mainly resulting from a faster dissociation rate (Fig. 1C). The greater structural instability of XBB.1.5 S(P2), evidenced by the lower T_m_, may account for the lower apparent ACE2 binding potency. Shedding and dissociation of the cleaved S1 and S2 following ACE2 binding, which likely occurs more readily in the less stable XBB.1.5 S(P2), could also contribute to the apparent dissociation, resulting in the faster *k*_off_ observed for XBB.1.5 S(P2) compared to the WT S(P2). These features of the XBB.1.5 S and its components could translate into increased fusion efficiency and account, in part, for the significant growth advantage this Omicron lineage gained over prior Omicron lineages. The selective advantage of one conformation versus another, however, remains unclear and raises questions about the optimal positioning of the RBD to best engage the human ACE-2 receptor, while potentially altering the exposure of key regions of the S protein to better escape host immunity.

The observed differences in the N-glycosylation pattern of XBB.1.5 S(P2) compared to the WT S also highlight that the evolution of SARS-CoV-2 is not only driven by amino acid changes and resulting structural conformations but potentially by other post-translational modifications that, for some virus fusion glycoproteins, serve to mask epitopes from antibody recognition. The structural analyses described here may thus inform an understanding of the evolutionary trajectory of SARS-CoV-2, in the context of different lineages, and in relation to other coronaviruses.

Although a correlate of protection for COVID-19 has not been definitively established, neutralizing antibody titers have trended closely with estimates of vaccine efficacy and effectiveness^30–32^. Neutralizing antibody responses observed in preclinical animal models have also associated with neutralization trends in clinical studies^33^. Therefore, assessment of the elicited neutralizing response by the variant-adapted vaccines was a paramount objective of the vaccine characterization. When administered as a booster dose or as a primary series in mice, the Omicron XBB.1.5-adapted BNT162b2 vaccine elicited superior neutralizing activity against XBB.1.5 and related XBB sublineage pseudoviruses, including the previously dominant EG.5.1 and HV.1 strains, compared to that elicited by the bivalent WT + BA.4/5 vaccine. The data support the conclusion that variant-adapted vaccines offer the ability to maintain optimal immune responses against evolving, circulating SARS-CoV-2 strains.

The more recently emerged Omicron BA.2.86 derivative JN.1 lineages, descendants of Omicron BA.2, have approximately sixty and thirty differences in the S amino acid sequence compared to the WT strain and Omicron XBB.1.5 sublineage, respectively. A sequence change of this magnitude has not been observed since the original emergence of Omicron BA.1, which contained approximately thirty amino acid changes relative to Delta, the prior VOC. Despite these sequence changes, the monovalent Omicron XBB.1.5-adapted booster vaccine sera neutralized BA.2.86 to a similar degree as other XBB sublineages, with improved responses over bivalent WT + Omicron BA.4/5 vaccine booster vaccine sera. In contrast, in a naive background, 2 doses of either the XBB.1.5-adapted or bivalent WT + BA.4/5-adapted vaccine conferred similarly low neutralizing activity against the BA.2.86 pseudovirus. The bivalent XBB.1.5 + BA.4/5 vaccine elicited slightly higher BA.2.86 neutralizing titers compared to the other formulations, suggesting a potentially broader coverage of the antigenic space inclusive of where Omicron BA.2.86 resides.

The large discrepancy in BA.2.86 neutralization between the booster and primary series studies indicates that the genetic sequence divergence of this lineage translates into an immunologic difference in a naive background but does not confer immune escape when the host has multiple prior exposures to antigens that broadly cover the SARS-CoV antigenic space. These data, therefore, demonstrate a significant antigenic distance of this new lineage from preceding ones, though that distance is rendered less important in a population with a diversity of prior immune experience. To date, more than 200,000 sequences of the BA.2.86 and JN.1 lineages and their derivatives (*i.e*., BA.2.86.1, JN.1.7, KP.2) have been deposited into GISAID since the first confirmed BA.2.86 case. BA.2.86 and JN.1 remain designated as variants of interest (VOI) by the World Health Organization due to the substantial amino acid changes in its S protein^34^. However, no sublineage from the BA.2.86 or JN.1 cluster has been reported to cause an increase in COVID-19 disease severity or deaths^35–41^.

The variant-adapted vaccines evaluated in this study, including the monovalent Omicron XBB.1.5 formulation, elicited robust Th1-type CD4^+^ and IFN-γ-secreting CD8^+^ T cell responses against S peptide pools representing the FL S of WT, Omicron BA.4/5 and Omicron XBB.1.5. These findings are consistent with observed trends for multiple variants where antigenic drift, and even major shifts, to new lineages and sublineages do not substantially erode previously established T cell-mediated immunity^42,43^. The likely consequence of a maintained cellular immune response is more durable effectiveness against severe clinical outcomes^44^. We did not examine T cell responses to BA.2.86 in this study; however, we anticipate similar findings as reported here based on prior assessments showing polyepitopic T cell responses against SARS-CoV-2 variants^21,45^.

The findings reported here demonstrate that the monovalent Omicron XBB.1.5-adapted BNT162b2 vaccine encodes a prefusion stabilized S(P2) protein that tightly binds the ACE-2 receptor, maintains a relatively open and flexible conformation, and confers optimal immune responses against contemporaneous SARS-CoV-2 strains. Strengths of this study include the booster immunogenicity study design, which aims to approximate the vaccine-experienced background of the BNT162b2-vaccinated population by pre-exposing animals to both the original monovalent WT vaccine and bivalent WT + BA.4/5 vaccine. The robust neutralizing response to the vaccine-encoded lineages to which the animals were exposed prior to receiving the XBB.1.5 vaccine indicates that immune imprinting plays a role in the de novo responses elicited by the updated vaccine, as has been noted in other studies^46,47^.

Limitations of this study include the inability to faithfully recapitulate the entire spectrum of immune experience, such as the hybrid immunity gained from prior SARS-CoV-2 infection and vaccination. This immune background likely reflects the majority of the vaccinated population, as seroepidemiology studies show that most individuals have experienced SARS-CoV-2, even among pediatric cohorts^48^. It was still important to evaluate the Omicron XBB.1.5-adapted vaccine in an immune naive setting, as there remains a steady proportion of individuals, primarily among the youngest pediatric population, who have not yet been exposed to SARS-CoV-2 through infection or vaccination. Although the present study does not include data from a SARS-CoV-2 animal challenge model, infection in animal models poorly recapitulate the more severe forms of human disease^49^, and vaccine-elicited neutralizing responses in preclinical immunogenicity models have trended closely with observed real-world vaccine effectiveness in humans^33^.

The aggregate data reported here provide a basis for expecting a robust immune response in humans, indicative of a reduction from severe disease outcomes such as hospitalization and death against XBB sublineage infections and resulting COVID-19 disease from ongoing clinical studies (*e.g.*, NCT05997290). Preclinical data have reliably predicted responses in humans to vaccination throughout the lifecycle of the original monovalent WT and variant-adapted BNT162b2 vaccines. These types of data now form the basis for regulatory authorizations and approvals of updated formulations, including the bivalent WT + Omicron BA.4/5 vaccine in 2022, and more recently, the monovalent Omicron XBB.1.5 vaccine. COVID-19 epidemiology and immunology continue to be dynamic; as such, safe and effective vaccines will need to keep pace by remaining adaptable to ensure rapid approval and broad access to at-risk populations.

## Methods

### Expression and purification of FL S(P2) and RBD proteins

In brief, protein sequences of the Omicron XBB.1.5 sublineage and WT (Wuhan-Hu-1) FL S(P2) encoded by BNT162b2 were used to generate a construct containing a C-terminal TwinStrep tag to facilitate affinity purification and were cloned into a pcDNA3.1(+) vector for expression. The RBD of each FL S(P2) (Omicron XBB.1.5 and WT) was expressed as secreted protein and purified via the engineered C-terminal affinity tag. Both FL S and RBD protein expressions were conducted in Expi293F cells (ThermoFisher Scientific) grown in Expi293 medium.

#### Expression and purification of FL S(P2)

Expression of proteins was carried out in Expi293F cells (Thermo Fisher Scientific) grown in Expi293 medium. Cells were transiently transfected with S or RBD protein expression constructs in the pcDNA3.1(+) vector. Expression was conducted at 37 °C for 24 hours before adding Expifectamine enhancers (Thermo Fisher Scientific). After addition of enhancers, the temperature was dropped to 32° C and expression was allowed for another 48–72 hours before collecting. A modified protocol of procedures described by Zhang et al.^50^ was used for purification of the SARS-CoV-2 FL S(P2). Briefly, the transfected cells were lysed in a solution containing Buffer A (100 mM HEPES pH 8.0, 150 mM NaCl, 1 mM EDTA), 1% (w/v) *n*-dodecyl-*β*-D-maltopyranoside (DDM, Anatrace), EDTA-free complete protease inhibitor cocktail (Roche), and Pierce Universal Nuclease (Thermo Fisher) at 4 °C for 1 h. After a clarifying spin at 40,000 ´ g for 45 min, the supernatant was filtered with 0.2 mm filter (Nalgene 78018-24, 1 LL) before batch bound onto StrepTactin HP resin (Cytiva) equilibrated with the lysis buffer at 4 °C for 1 h. Resin was collected by centrifugation at 1000 ´ g and loaded onto EconoColumn (Bio-Rad) for gravity flow purification. The column was washed with Buffer A containing 0.5% DDM, 10 mM ATP, and 10 mM MgCl_2_, followed by additional washes with Buffer A and gradually reduced concentrations of DDM (0.5–0.02%). FL S(P2) was eluted with Buffer A containing 0.02% DDM and 5 mM d-Desthiobiotin. The protein was further purified by size exclusion chromatography (SEC) on a Superose 6 10/300 column (Cytiva) in a buffer containing 25 mM Tris pH 7.5, 150 mM NaCl, 1 mM EDTA, and 0.02% DDM. DDM-purified FL S(P2) was eluted as a single peak over SEC. FL S(P2) protein from the SEC peak fractions were analyzed by denaturing PAGE using a 4–15% Criterion TGX Stain-Free Gel (Bio-Rad, Supplementary Fig. 1), and used in thermostability (T_m_), biolayer interferometry (BLI), mass spectrometry and cryogenic electron microscopy (cryo-EM) experiments.

#### Expression and purification of RBD

The RBDs were expressed using Expi293F cells (Thermo Fisher Scientific) grown in Expi293 medium transiently transfected with the RBD expression constructs in pcDNA3.1(+) vector. The RBD constructs contain an N-terminal S protein leader peptide and coding regions from 324–531 (Omicron XBB.1.5) and 327–528 (WT ancestral strain), respectively, followed by a C-terminal affinity tag as indicated in Supplementary Table 1. Expression was conducted at 37 °C for 120 hours before the proteins were collected from cell culture medium. The affinity tagged RBDs were purified on affinity purification columns first, subsequently on Superdex200 gel filtration column (Cytiva), and stored in a buffer containing 100 mM Tris pH7.5, 150 mM NaCl, and 10% glycerol. RBD was expressed as secreted protein and purified via the engineered C-terminal affinity tag.

### Stability of WT and Omicron XBB.1.5 FL S(P2) by Thermal Shift Assay (TSA)

Stability of FL S(P2) proteins was measured by Tycho NT.6 (NanoTemper, firmware version: 1.10.3) and the data were analyzed by the Tycho NT.6 software (version: 1.3.2.880). In brief, a 10 mL solution containing 0.35 mg/mL of protein was loaded into a capillary tube and the ratio of tryptophan fluorescence at emission wavelengths of 350 nm over 330 nm and was measured while ramping the temperature from 35 °C to 95 °C using the pre-programmed protocol of the instrument. The inflection temperature for each thermal melting curve was reported by the Tycho NT.6 software.

### Binding kinetics of purified FL S(P2) protein and RBD to immobilized human ACE-2-PD

FL S(P2), with a C-terminal TwinStrep tag expressed in Expi293F cells, was detergent solubilized and purified by affinity and size exclusion chromatography. The peak fraction of the purified FL S(P2) and isolated RBD proteins of Omicron XBB.1.5 and WT strains were assessed by biolayer interferometry (BLI) binding to immobilized human ACE-2-PD on an Octet RED384 (FortéBio) at 25 °C in a running buffer that comprised 25 mM Tris pH 7.5, 150 mM NaCl, 1 mM EDTA, and 0.02% DDM, identical to the protein purification buffers. The highest concentration assessed for both FL S(P2) and RBD was 300 nM, with three additional three-fold dilutions. BLI data were collected with Octet Data Acquisition software (version 10.0.0.87) and processed and analyzed using FortéBio Data Analysis software (version 10.0). Binding curves were reference subtracted and fit to a 1:1 Langmuir model to determine binding kinetics and affinity.

### Cryo-EM of Omicron XBB.1.5 FL S(P2)

Purified Omicron XBB.1.5 FL S(P2) was applied to glow discharged Quantifoil R1.2/1.3 200 mesh gold grids and blotted using a Vitrobot Mark IV (ThermoFisher Scientific) before being plunged into liquid ethane cooled by liquid nitrogen. Datasets were collected and analyzed as depicted in Supplementary Fig. 3. The purified sample in DDM at 5.0 mg/mL were applied onto the glow discharged Quantifoil R1.2/1.3 200 mesh gold grids and blotted using a Vitrobot Mark IV (ThermoFisher Scientific). A data set of 6690 movies was recorded using EPU from a Titan Krios G2 transmission electron microscope operating at 300 keV equipped with a Falcon 4i direct electron detector and Selectris Energy Filter (ThermoFisher Scientific). Each movie was collected in counting mode with a pixel size of 0.75 Å/pixel, 10 eV slit and a defocus range of −0.6 μm to −2.6 μm for a total dose of 40.0 e^−^/Å^2^. Each data set was imported and processed in CryoSPARC v4.2.1. All movies were adjusted with patch motion correction and patch CTF estimation. Templates were generated from 2D class averages after automative particle picking by blob picker. These 2D class average templates were used for template-based autopicking to pick particles for the rest of the data processing.

From Template Picker, 1,862,402 particles were autopicked and extracted with a box size of 540 pixels. Iterative 2D classification were carried out to select particles with high resolution views of the spike protein (3131,229 particles). Three initial models were generated using all 131,229 particles in ab initio reconstruction resulting in only one map containing 91,663 particles that resemble a spike protein with 1-RBD-up. Heterogeneous refinement of the selected particles resulted in only 1-RBD-up structures. Therefore, all 91,663 particles were subjected to homogeneous refinement, followed by non-uniform refinements, which gave the final 1-RBD-up structure with an overall resolution of 2.98 Å. All refinement steps were done with C1 symmetry. The final resolution was calculated from the Fourier Shell Correlation (FSC) curve from the resolution at the 0.143 FSC cutoff.

A model of the Omicron spike protein structure (PDB: 7TGW)^51^ was fitted into the final cryo-EM structure and was used as a guide for modeling. The atomic model was built in COOT^52^ and refined using Phenix real space refinement^53^. The EM density for the RBD in the up position was weakly resolved. Therefore, the RBD was docked into the EM density and rigid body fitted without side chains unless there were clear side chain densities. The final model including the RBDs were refined in Phenix (version 1.20-4459-0000).

### Mass spectrometry characterization of N-linked glycosylation

Mapping of N-linked glycosylation sites was conducted on recombinant purified Omicron XBB.1.5 S(P2) following precipitation using ice cold acetone and incubated overnight at −20 °C. The protein was pelleted, dissolved in 8 M urea, and reduced and alkylated prior to proteolytic digestion. Protein samples were digested in three batches using either trypsin, trypsin/Glu-C, or chymotrypsin. Digested peptide pools from all three reactions were subjected to mass spectrometry analysis to achieve a desired sequence coverage (~92%). Peptides were separated from remaining enzymes using a Microcon-10kDa centrifugal filter (MRCPRT010). The supernatant was collected and lyophilized to dryness. For N-linked analysis, digested samples were reconstituted in O18 water. PNGase F and O-glycosidase were added to remove N-linked and O-linked glycosylation.

The treated digests were analyzed on a Thermo QExactive Oribitrap Mass Spectrometer with an EZ-NanoSpray Source and an EZ-nLC 1200. The peptides were chromatographically separated prior to in-line mass spectrometry analysis with a flow rate of 2 mL/min. The samples were also analyzed on a Thermo Fusion Tribrid Mass Spectrometer outfitted with an EZ-nLC 1200 to perform peptide separations. The system was operated in direct injection mode and the peptides were chromatographically separated prior to in-line mass spectrometry analysis with a flow rate of 450 nL/min. Matrix Science MASCOT and Thermo Freestyle were used for data analysis. The N-linked data was searched with the following variable modifications: Deamidated (NQ), Deamidated:18 O(1) (NQ), HexNAc (N), Oxidation (M).

### Animal ethics

All murine experiments were performed at Pfizer, Inc. (Pearl River, NY, USA), which is accredited by the Association for Assessment and Accreditation of Laboratory Animal

Care (AAALAC). All procedures performed on animals were in accordance with regulations and established guidelines and were reviewed and approved by an Institutional Animal Care and Use Committee or through an ethical review process.

### BNT162b2 mRNA XBB.1.5 vaccine modification and formulation

The XBB.1.5 adapted vaccine encodes the S(P2) of XBB.1.5 (GISAID EPI_ISL_16292655) on the BNT162b2 RNA backbone. Purified nucleoside-modified RNA was formulated into lipid nanoparticles by mixing together an organic phase lipid mixture with an RNA aqueous phase, and subsequently purifying the mix to yield a lipid nanoparticle composition similar to one previously described^54^.

### Immunogenicity in BNT162b2-experienced mice

Female BALB/c mice (10 per group, age 6–8 weeks; Jackson Laboratory) were vaccinated and bled as shown in Supplementary Fig. 4A. In brief, mice were vaccinated intramuscularly with a 2-dose series (Day 0, 21) of a 0.5 µg dose level of original BNT162b2 WT vaccine, followed by a 3rd dose booster (Day 105) of bivalent WT + Omicron BA.4/5 vaccine, and a 4th dose booster (Day 134) of either monovalent Omicron XBB.1.5, monovalent Omicron BA.4/5, bivalent Omicron XBB.1.5 + BA.4/5 or bivalent WT + Omicron BA.4/5 sublineage-modified vaccines. Bivalent formulations contained equal quantities of each mRNA (0.25 µg each) and a total dose level of 0.5 µg. A control group of ten mice received saline injections according to the same schedule in place of active vaccines. A total volume of 50 µL of vaccine or saline was administered intramuscularly to the upper outer hind leg for each animal. Animals and injection sites were observed immediately after vaccination. Sera were collected for evaluation of pseudovirus neutralizing antibody responses prior to the 4th dose (Day 134) and at the final post-vaccination timepoint (Day 160). Spleens were also collected at Day 160 to evaluate cell-mediated immune responses.

### Immunogenicity in naive mice

Female BALB/c mice (10 per group, age 10–12 weeks; Jackson Laboratory) were vaccinated and bled according to the illustration in Supplementary Fig. 4B. In brief, mice were vaccinated intramuscularly on Days 0 and 21 with either monovalent Omicron XBB.1.5, monovalent Omicron BA.4/5, bivalent Omicron XBB.1.5 + BA.4/5 or bivalent WT + Omicron BA.4/5-adapted vaccines. The control group received saline injections according to the same schedule as active vaccine groups. Sera and spleens were collected 28 days after the second dose (day 49) for evaluation of pseudovirus neutralizing antibody responses and cell-mediated immune responses, respectively.

### Pseudovirus neutralization assay

Pseudovirus stocks were generated in HEK-293T cells (ATCC, ref.# CRL-3216) using SARS-CoV-2 spike plasmid DNA and vesicular stomatitis virus (VSV; VSVΔG(G)-GFP virus: Kerafast, ref.# EH1019-PM). Serial dilutions of heat-inactivated murine sera (3-fold) were incubated with pseudovirus (VSVΔG(G)-GFP expressing SARS-CoV-2 S protein) for 1 h at 37 °C before inoculating confluent Vero (ATCC, ref.# CCL81.2) cell monolayers in 96-well plates. Fluorescent virus-infected foci were detected 19–21 h after inoculation with an anti–VSV pAb (Imanis Life Sciences, ref# REA005) and Alexa488-conjugated secondary antibody (Invitrogen, ref# A-11008) and enumerated using a CTL Immunospot Analyzer (Cellular Technology Limited). A 50% neutralization titer (NT_50_) was calculated as the last reciprocal serum dilution at which 50% of the virus is neutralized compared to wells containing virus only. Each serum sample dilution was tested in duplicate. The assay titer range was 20 to 43,740. Any serum sample that yielded a titer >43,470 was prediluted and repeated to extend the upper titer limit; sera that failed to neutralize at the lowest serum dilution (1:20) were reported to have a neutralizing titer of 20 (lower limit of detection, LLOD). VSV-based pseudoviruses used in the assay expressed the S protein from the following SARS-CoV-2 variants: WT (Wuhan-Hu-1, ancestral strain), BA.4/5, XBB.1.5, XBB.1.16, XBB.1.16.1, XBB.2.3, EG.5.1, HV.1 and BA.2.86. Amino acid sequence alignments for all tested pseudoviruses are provided in Supplementary Fig. 8.

### T-cell response assay

In brief, antigen-specific T cell responses were analyzed from murine splenocytes with a flow cytometry-based intracellular cytokine staining (ICS) assay, comparing unstimulated (DMSO) response to those observed in splenocytes after stimulation with a peptide library. Freshly-isolated splenocytes (2 × 10^6^ cells/well) were cultured in cRPMI with media containing DMSO only (unstimulated) or specific amino acid (aa) peptide libraries (15aa, 11aa overlap, 1 to 2 µg/mL/peptide) representing the S amino acid sequences of the original SARS-CoV-2 Wuhan strain (WT), Omicron BA.4/5, and Omicron XBB.1.5 sublineages, separately (JPT, catalog #s PM-SARS2-SMUT10-2, PM-SARS2-SMUT15-1, PM-WCPV-S-1), for 5 h at 37 °C in the presence of protein transport inhibitors, GolgiPlug and GolgiStop. Following stimulation, splenocytes were incubated with fluorescent-conjugated antibodies to the surface proteins CD19, CD3, CD4 and CD8 (25 ± 5 min at RT) followed by fixation and permeabilization and staining for CD154 (CD40L), IFN-γ, TNF-α, IL-2, IL-4, and IL-10 (25 ± 5 min at RT). Ebioscience fixable viability dye eFluor 506 was used exclude dead cells. After staining, the cells were washed and resuspended in flow cytometry (FC) buffer (2% FBS in PBS). Samples were acquired on a BD LSR Fortessa flow cytometer with BD FACSDiva software. Acquired data files were analyzed using BD FlowJo™ (version 10.8). Results are background (media-DMSO) subtracted and shown as percentage of CD154^+^ cytokine-expressing CD4^+^ T cells and CD8^+^ T cells. The Tcell gating strategy is shown in Supplementary Fig. 7.

### Animal blood collection and splenocyte isolation

For interim blood draws, mice were bled via the submandibular route. Approximately 150 μL of whole blood was collected dropwise directly into microtainer tubes containing serum separators. For terminal blood draws, the entire available blood volume was collected via cardiac puncture. At each study end, mice were euthanized under a surgical plane of isoflurane and cervical dislocation was performed as a secondary method to confirm death. Submandibular bleeds and vaccinations were not done under sedation. At all blood collection time points, blood tubes remained at room temperature (RT) for at least 30 min prior to centrifuging at 12,300 ´ g for 3 min. Each serum sample was aliquoted and heat inactivated at 56 °C for 30 min. Samples were stored at −80 °C after testing.

For flow cytometry of murine splenocytes, spleens were collected from five mice per group at the final time point for each study. Spleens were placed in a 100 µm cell strainer (BD Falcon) immersed in 7 mL of complete RPMI (cRPMI: 10% FBS/RPMI; Pen-Strep; Sodium pyruvate) per mouse per well of a 6-well plate. The plates were maintained on ice during transit and before processing for single cell suspension. Spleens were homogenized, subjected to RBC lysis, and passaged through a cell strainer to remove RBCs and clumps.

### Statistical analysis

Mouse immunogenicity data were analyzed using SAS version 9.4. All statistical analyses were performed using ANOVA on log-transformed data. Comparisons were made on mouse sera across pseudoviruses at the last post-vaccination timepoint of each study with Dunnett’s test for multiple comparisons. For intergroup comparisons, the bivalent WT + Omicron BA.4/5 vaccine group was the reference; for intragroup comparisons (pseudoviruses within a vaccine group), the Omicron XBB.1.5 was the reference. All tests were two-tailed. A *p*-value of less than 0.05 was considered statistically significant.

## Supplementary information

Supplementary information

## Data Availability

The final full-length XBB.1.5 S(P2) cryo-EM density map and model are deposited in the Electron Microscopy Data Bank (EMDB) and Protein Data Bank (PDB) under accession codes EMD-42524 and PDB ID 8USZ, respectively. All other data are contained within the manuscript and supplementary material files.
