# Supplementary material for: Preclinical characterization of the Omicron XBB.1.5-adapted BNT162b2 COVID-19 vaccine
Source: NPJ Vaccines. 2024 Nov 20;9:229. doi: 10.1038/s41541-024-01013-9 (PMC11579292; doi:10.1038/s41541-024-01013-9)
Supplement: Supplementary file 1 — Supplementary information [file 41541_2024_1013_MOESM1_ESM.pdf]

Supplementary Materials for

Preclinical Characterization of the BNT162b2 Omicron XBB.1.5-Adapted COVID-19 Vaccine

Modjarrad et al.

Contents

Supplementary Figure 1. SDS-PAGE of DDM-Solubilized and Purified S(P2) WT and Omicron XBB.1.5 S(P2) Proteins 2

Supplementary Figure 2. Glycosylation Pattern of the XBB.1.5 S(P2) Protein 3

Supplementary Figure 3. Cryo-EM workflow for construct pSB6534 4

Supplementary Figure 4. Schema for BNT162b2 variant-modified vaccine mouse immunogenicity studies 5

Supplementary Figure 5. Baseline pseudovirus neutralization titers (NT50) prior to BNT162b2 variant-adapted vaccine booster immunization in BNT162b2-experienced mice. 6

Supplementary Figure 6. Geometric mean fold rise in pseudovirus neutralization titers (NT50) from pre- to post-4th dose with a BNT162b2 variant-adapted vaccine in vaccine-experienced mice 7

Supplementary Figure 7. Gating strategy for intracellular cytokine staining flow cytometry analysis of T cell responses 8

Supplementary Figure 8. SARS-CoV-2 S amino acid sequence differences across lineages and sublineages for generated pseudoviruses 9

Supplementary Table 1. Expression Constructs of FL S(P2) and RBD proteins 10

Supplementary Table 2. Cryo-EM Data Collection, Processing and Refinement Statistics 11

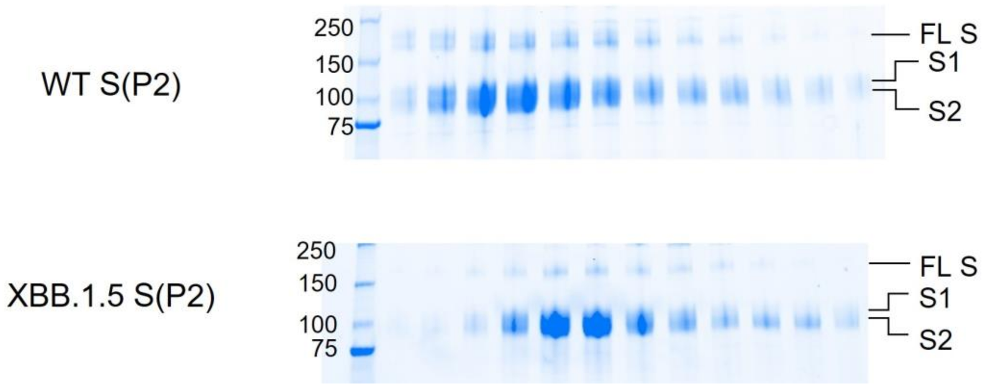

Supplementary Figure 1. SDS-PAGE of DDM-Solubilized and Purified S(P2) WT and Omicron XBB.1.5 S(P2) Proteins. SDS-PAGE of the SEC fractions from 11.5 mL to 15 mL of the WT S(P2) (upper panel) and 11 mL to 14.5 mL of FL Omicron XBB.1.5 S(P2) (lower panel).

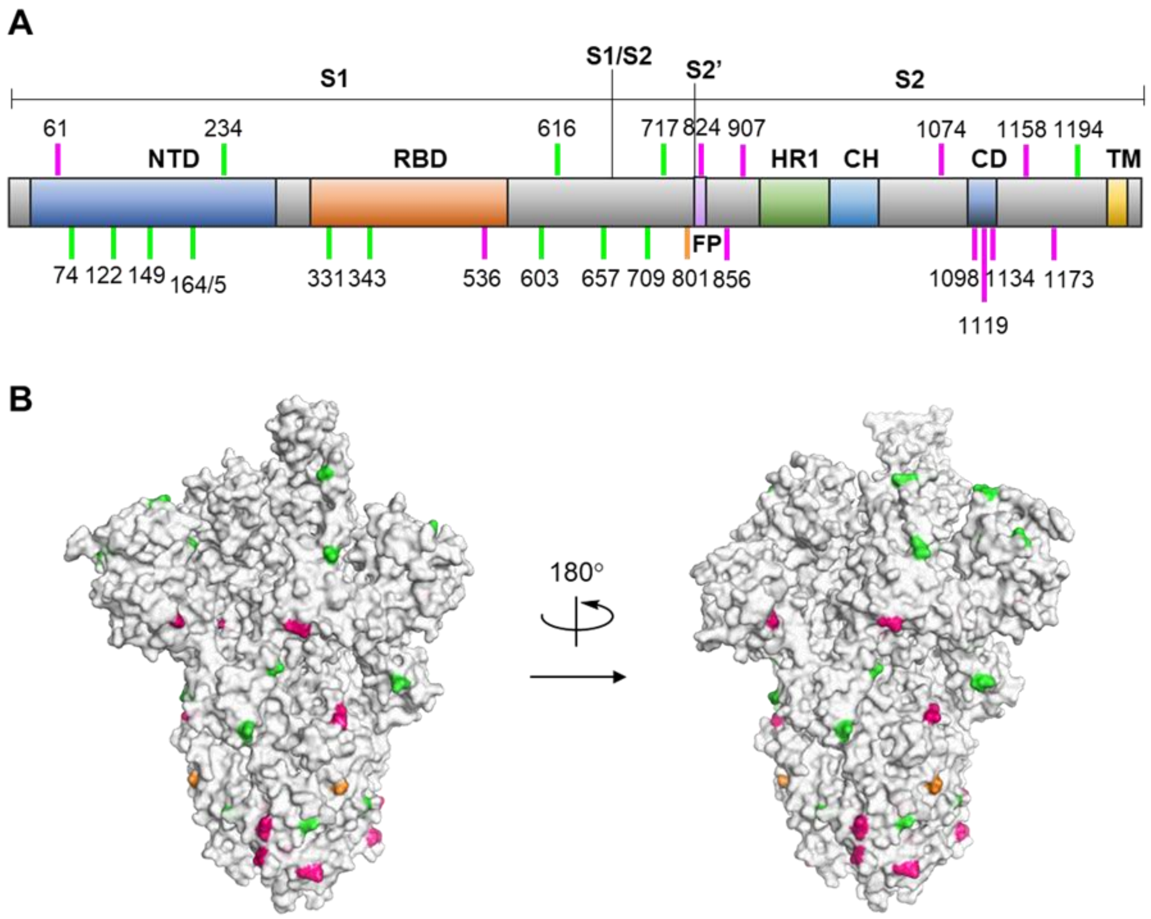

Supplementary Figure 2. Glycosylation Pattern of the XBB.1.5 S(P2) Protein. A. Schematic representation of the Omicron XBB.1.5 S glycoprotein. The positions of N-linked glycosylation observed by mass spectrometry studies are shown as bar lines. Glycan sites are colored according to glycan content: green (75 to 100%), orange (50 to 75%) and pink (0 to 50%). Protein domains are illustrated: N-terminal domain (NTD), receptor binding domain (RBD), fusion peptide (FP), heptad repeat 1 (HR1), central helix (CH), connector domain (CD), and transmembrane domain (TM). S protein S1 subunit, S2 (subunit after furin cleavage site to C-terminus) and S2' (subunit after TMPRSS2 cleavage site within the fusion peptide of Spike to C-terminus) boundaries are also illustrated. **B.** Structure-based mapping of N-linked glycans on the Omicron XBB.1.5 FL S(P2) Cryo-EM structure. Glycans are not modeled. Only glycosylated asparagine residues are highlighted and colored according to glycan content defined in **A**.

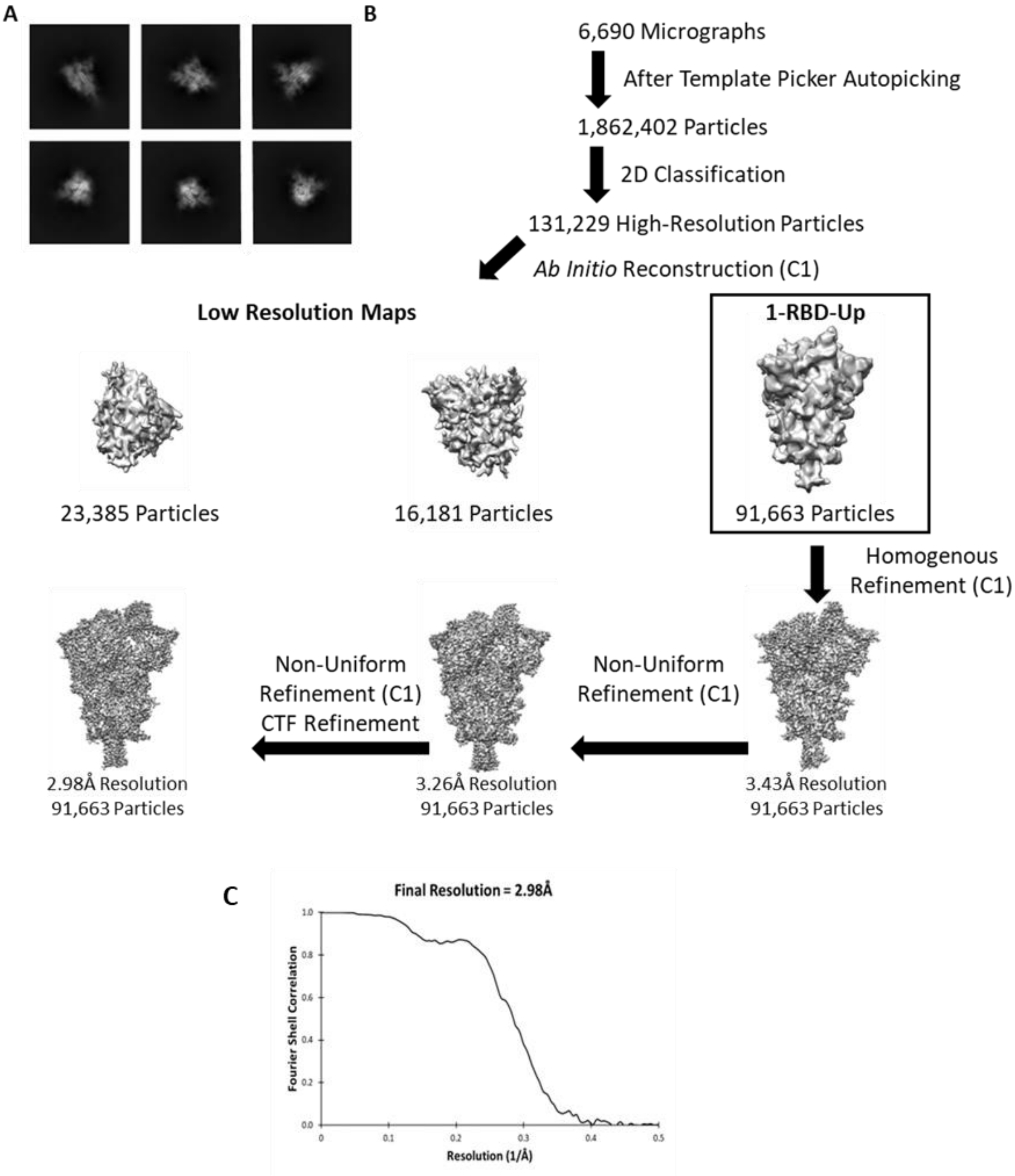

Supplementary Figure 3. Cryo-EM workflow for construct pSB6534. A. Representative two-dimensional class averages of Omicron XBB.1.5 FL S(P2). **B.** Cryo-EM data processing flowchart revealed 1-RBD-up as the primary S protein conformation. Particle number, Fourier shell correlation curve and corresponding resolution for the final map are indicated. **C.** Fourier shell correlation (FSC) curve indicates an overall nominal resolution of 2.98 Å using the gold standard FSC = 0.143 criterion.

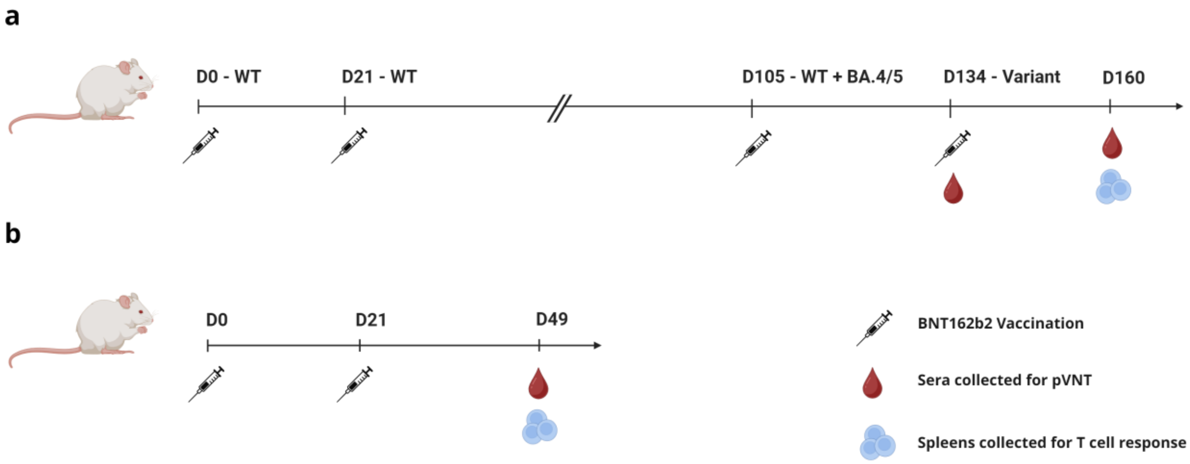

Supplementary Figure 4. Schema for BNT162b2 variant-modified vaccine mouse immunogenicity studies. A. In the booster study, female BALB/c mice were administered a two-dose series of the original monovalent BNT162b2 (WT) vaccine (Day 0, 21), followed by a third dose with the bivalent WT + BA.4/5 BNT162b2 vaccine (Day 105), and a 4th dose (Day 134) with one of the following variant-modified BNT162b2 vaccines: monovalent Omicron BA.4/5, bivalent WT + Omicron BA.4/5, monovalent Omicron XBB.1.5 or bivalent Omicron XBB.1.5 + Omicron BA.4/5. Sera for pseudovirus neutralization test (pVNT) were collected just prior to the final vaccination (Day 134) and the last post-vaccination timepoint (Day 160). **B.** In the primary series study, female BALB/c mice (10 per group) were administered a two-dose series (Day 0, 21) of one of the following variant-modified BNT162b2 vaccines: monovalent Omicron BA.4/5, bivalent WT + Omicron BA.4/5, monovalent Omicron XBB.1.5 or bivalent Omicron XBB.1.5 + Omicron BA.4/5. Sera for the pVNT were collected at 2 weeks after the second vaccination (Day 49). In both **A** and **B**, mice were vaccinated in groups of 10, spleens were collected at the final post-vaccination timepoints, and the total dose level for each vaccine formulation administered was 0.5 µg. Created in BioRender. D'Arco, C. (2024) BioRender.com/v29f815

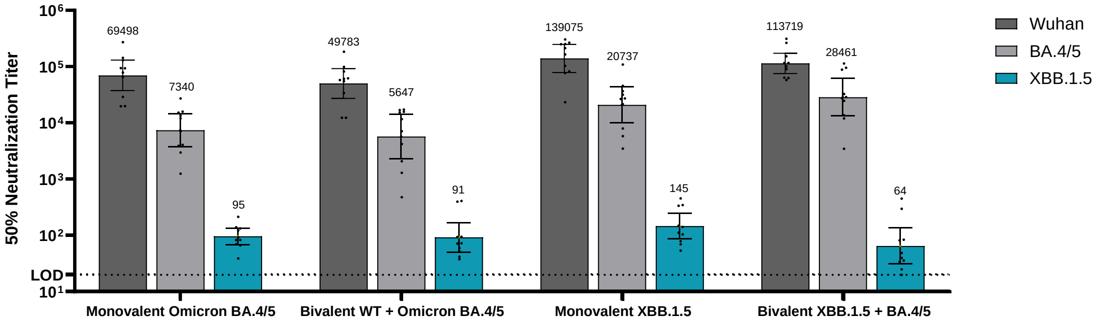

Supplementary Figure 5. Baseline pseudovirus neutralization titers (NT50) prior to BNT162b2 variant-adapted vaccine booster immunization in BNT162b2-experienced mice. Female BALB/c mice (10/group) previously vaccinated with two doses of monovalent original BNT162b2, and one subsequent dose of bivalent BNT162b2 (WT + Omicron BA.4/5) received a single intramuscular dose of one of the following variant modified BNT162b2 vaccines: monovalent Omicron BA.4/5, bivalent WT + Omicron BA.4/5, monovalent Omicron XBB.1.5 or bivalent Omicron XBB.1.5 + Omicron BA.4/5. All vaccine formulations contained a total dose of 0.5 µg. Serum neutralizing antibody responses were assessed in a pseudovirus neutralization assay for the pre-fourth dose timepoint against the Wuhan reference strain, and the Omicron sublineages BA.4/5 and XBB.1.5. 50% pseudovirus neutralization titers are shown as geometric mean titers (GMT) with 95% CI of 10 mice per vaccine group. The limit of detection (LOD) is the lowest serum dilution, 1:20. Each point represents an individual animal.

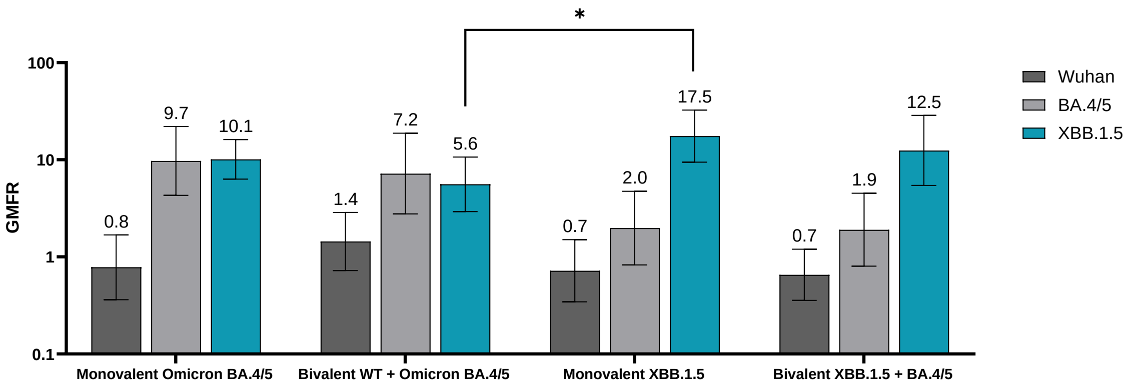

Supplementary Figure 6. Geometric mean fold rise in pseudovirus neutralization titers (NT50) from pre- to post-4th dose with a BNT162b2 variant-adapted vaccine in vaccine-experienced mice. Female BALB/c mice (10/group) that were previously vaccinated with two-doses of original monovalent BNT162b2 (WT) vaccine , and one subsequent dose of bivalent WT + Omicron BA.4/5 vaccine received a single intramuscular booster dose of one of one of the following variant-modified BNT162b2 vaccines: monovalent Omicron BA.4/5, bivalent WT + Omicron BA.4/5, monovalent Omicron XBB.1.5 or bivalent Omicron XBB.1.5 + Omicron BA.4/5. All vaccine formulations contained a total dose of 0.5 µg. Serum neutralizing antibody responses were measured by a pseudovirus neutralization assay. The fold rise in geometric mean neutralizing titers (GMFR) from pre-fourth dose to one-month post-dose are shown with 95% CI for the Wuhan reference strain, Omicron BA.4/5 and XBB.1.5. The limit of detection (LOD) is the lowest serum dilution, 1:20. Statistical differences between vaccine groups were analysed by ANOVA (*$p<0.05$).

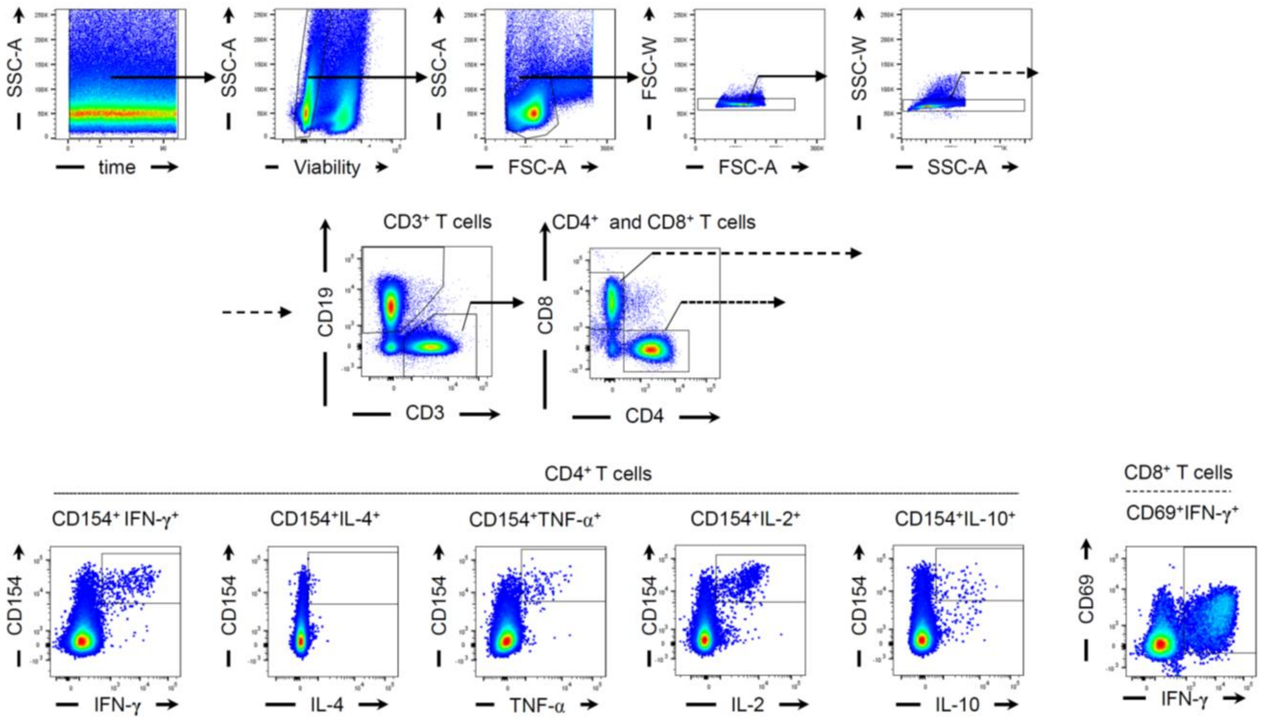

Supplementary Figure 7. Gating strategy for intracellular cytokine staining flow cytometry analysis of T cell responses. Flow cytometry gating strategy for identification of SARS-CoV-2 spike-specific T cells for different sublineages. (Upper row, left to right) Starting with events acquired with a constant flow stream and fluorescence intensity, viable cells, lymphocytes, and single events were identified and gated. Within singlet lymphocytes, CD19-negative CD3+ T cells were identified and gated into CD4+ and CD8+ T cells (middle row). Antigen-specific CD4+ T cells were identified by gating on CD154 and cytokine-positive cells. Activated CD8+ T cells were identified by gating on CD69 and cytokine-positive cells (bottom row). The antigen-specific cell frequencies were used for further analysis.

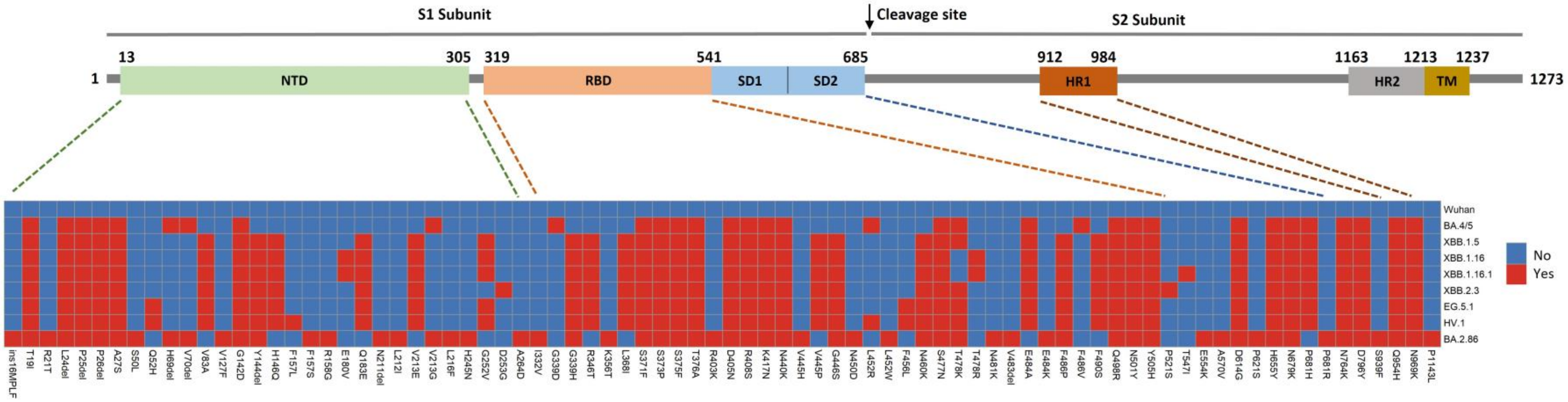

Supplementary Figure 8. SARS-CoV-2 S amino acid sequence differences across lineages and sublineages for generated pseudoviruses. Isolates metadata for each lineage was downloaded from GISAID (https://gisaid.org/) by filtering lineage name. Frequency of spike (S) protein amino acid difference relative to the ancestral strain within each lineage was calculated and a consensus S protein amino acid sequence list was generated by prioritizing sequence differences observed across more than 50% of sequences in the GISAID database. Consensus S protein amino acid sequence lists for all lineages were then aligned and plotted using R library heatmap. NTD – N-terminal domain; RBD-receptor binding domain; SD-subdomain 1; SD2-subdomain 2; HR1-heptad repeat 1; HR2-heptad repeat 2; TM-transmembrane domain.

Supplementary Table 1. Expression Constructs of FL S(P2) and RBD proteins

Code	Description	Mutations	Affinity Tag
pSB2782	S(P2) (WT)	K986P, V987P	C-terminal TwinStrep
pSB6534	S(P2) (Omicron XBB.1.5)	K986P, V987P, T19I, L24del, P25del, P26del, A27S, V83A, G142D, Y144del, H146Q, Q183E, V213E, G252V, G339H, R346T, L368I, S371F, S373P, S375F, T376A, D405N, R408S, K417N, N440K, V445P, G446S, N460K, S477N, T478K, E484A, F486P, F490S, Q498R, N501Y, Y505H, D614G, H655Y, N679K, P681H, N764K, D796Y, Q954H, N969K	C-terminal TwinStrep
pSB2661	RBD (WT)		C-terminal StrepII
pSB6876	RBD (Omicron XBB.1.5)	G339H, R346T, L368I, S371F, S373P, S375F, T376A, D405N, R408S, K417N, N440K, V445P, G446S, N460K, S477N, T478K, E484A, F486P, F490S, Q498R, N501Y, Y505H	C-terminal His´8

Supplementary Table 2. Cryo-EM Data Collection, Processing and Refinement Statistics

**Data Collection and Processing**	Omicron XBB.1.5 FL S(P2)
Magnification	130,000x
Voltage (kV)	300
Total Electron Exposure ($e^-/Å^2$)	40.0
Defocus Range (μm)	-0.6 to -2.8
Pixel Size (Å) - Counting Mode	0.750
Movies Recorded	6,690
Symmetry Imposed	C1
Initial Particle Images (no)	1,862,402
Final Particle Images (no)	91,633
Map Resolution at FSC=0.143 (Å)	2.98
**Refinement**	
Map sharpening *B* factor ($Å^2$)	-55.8
Model composition in the asymmetric unit	
Non-hydrogen atoms	21,179
Protein residues	2,758
B factors ($Å^2$)	
Protein	35.997
R.M.S. Deviations	
Bond lengths (Å)	0.004
Bond angles (°)	0.734
Validation	
MolProbity score	1.78
Clashscore	3.04
Poor rotamer (%)	2.71
Ramachandran Plot	
Favored (%)	94.83
Allowed (%)	5.17
Outliers (%)	0.00
